# The Potential Role of Aerobic Exercise-Induced Pentraxin 3 on Obesity-Related Inflammation and Metabolic Dysregulation

**DOI:** 10.1155/2017/1092738

**Published:** 2017-03-17

**Authors:** Aaron L. Slusher, Chun-Jung Huang, Edmund O. Acevedo

**Affiliations:** ^1^Department of Kinesiology and Health Sciences, Virginia Commonwealth University, Richmond, VA 23284, USA; ^2^Exercise Biochemistry Laboratory, Department of Exercise Science and Health Promotion, Florida Atlantic University, Boca Raton, FL 33431, USA

## Abstract

Obesity is defined as the excess accumulation of intra-abdominal body fat, resulting in a state of chronic, low-grade proinflammation that can directly contribute to the development of insulin resistance. Pentraxin 3 (PTX3) is an acute-phase protein that is expressed by a variety of tissue and cell sources and provides an anti-inflammatory property to downregulate the production of proinflammatory cytokines, in particular interleukin-1 beta and tumor necrosis factor alpha. Although PTX3 may therapeutically aid in altering the proinflammatory milieu in obese individuals, and despite elevated expression of PTX3 mRNA observed in adipose tissue, the circulating level of PTX3 is reduced with obesity. Interestingly, aerobic activity has been demonstrated to elevate PTX3 levels. Therefore, the purpose of this review is to discuss the therapeutic potential of PTX3 to positively regulate obesity-related inflammation and discuss the proposition for utilizing aerobic exercise as a nonpharmacological anti-inflammatory treatment strategy to enhance circulating PTX3 concentrations in obese individuals.

## 1. Introduction

Obesity is a state of chronic, low-grade proinflammation that derives from the excess infiltration and accumulation of neutrophils and resident macrophages within the adipose tissue [1–3]. This proinflammatory state directly contributes to the increased risk and pathology of obesity-related metabolic dysfunction, including insulin resistance and the subsequent development of type 2 diabetes mellitus [4, 5]. Alarmingly, adult obesity prevalence rates in the United States have significantly increased from 30.5% to 37.7% since the turn of the century [6], highlighting the need to identify potential therapeutic approaches that attenuate obesity-related proinflammatory profiles.

Counter to obesity-related inflammation, pentraxin 3 (PTX3) is an acute-phase protein that is induced in response to proinflammatory stimulation by a variety of cell types, including adipocytes, neutrophils, monocytes, and macrophages [7–9]. With obesity, PTX3 mRNA expression is elevated in adipose tissue and is positively associated with the mRNA expression of the proinflammatory cytokines interleukin-1 beta (IL-1*β*) and tumor necrosis factor alpha (TNF-*α*) [10, 11]. However, our laboratory and others have shown that circulating concentrations of PTX3 are reduced in obese individuals [11–13], suggesting that circulating PTX3 concentrations are dysregulated during obesity. Recent studies further demonstrate that PTX3 elicits the production of anti-inflammatory cytokines IL-10 and transforming growth factor beta (TFG-*β*) while downregulating the proinflammatory response [14–16]. Therefore, the purpose of this review is to discuss the potential factors contributing to obesity-related PTX3 dysregulation. Furthermore, this review will address the therapeutic potential of PTX3 to improve obesity-related inflammatory imbalance, and because of the reported elevations in PTX3 following aerobic exercise [12, 13, 17], this review will address the possibility of utilizing aerobic exercise as a nonpharmacological anti-inflammatory treatment strategy to enhance plasma PTX3 concentrations in obese individuals.

## 2. Structure and Function of Pentraxin Superfamily

Pentraxins are an evolutionarily conserved superfamily of proteins [18], identified by a 203 amino acid long “pentraxin domain” consisting of the His-x-Cys-x-Ser/Thr-Trp-x-Ser amino acid sequence in the carboxy-terminal region, where x is any amino acid [19–22]. Elucidation of the structural characteristics has led to the organization of pentraxins into two groups, the short and the long pentraxins, based on the N-terminal domain that is attached to the pentraxin domain [21].

### 2.1. Short Pentraxins

Short pentraxins consist of C-reactive protein (CRP) and serum amyloid P component (SAP). First identified in 1930, elevated concentrations of CRP, then termed fraction C, were observed in patients during the acute stage of pneumococcus infection and remained depending on the presence and severity of illness [23]. In 1941, MacLeod and Avery referred to CRP as an “acute-phase reactant” to describe its rapid accumulation in the blood following inflammatory challenge [24]. CRP and SAP are now known to be secreted primarily by the liver, and to lesser extents by leukocytes, following stimulation by the proinflammatory cytokine IL-6 [25–28]. Although baseline concentrations of CRP typically do not exceed 2-3 *μ*g/mL, up to 1000-fold increases in CRP are observed in response to a variety of adverse inflammatory conditions, whereas SAP concentrations remain relatively stable between 30 and 50 *μ*g/mL [29]. These findings have contributed to the utilization of CRP, but not SAP, as an indirect clinical marker of inflammatory disease status, particularly in cardiovascular disease and metabolic dysregulation [30].

### 2.2. Long Pentraxins

Long pentraxins contain longer N-terminal domains compared to their short pentraxin counterparts and include, among others, pentraxin 3 (PTX3), PTX4, and neutronal pentraxins 1 and 2. Of these, PTX3, also known as TNF-stimulated gene (TSG-14), was the first to be discovered in 1990 and is the most well studied. PTX3 has a molecular weight of ~42 kDa, is located on chromosome 3 (q25), consists of three exons, and contains a unique 178 amino acid long sequence which is structurally unique compared to all other proteins [21, 31]. PTX3 was first documented as an acute-phase reactant in 1992 [21] and is now known to be evolutionarily conserved between murine and human species, as protein sequences encoded by exon 1 and exon 2 share 93 and 81% structural homology, respectively [32]. Furthermore, Breviario et al. [21] demonstrate that vascular endothelial cell expression of PTX3 mRNA is increased within 1 hour and peaks between 2 and 6 hours following stimulation with the proinflammatory cytokines IL-1*β* and TNF-*α*, but not IL-6. Similarly, Lee et al. [31] observed peak PTX3 mRNA expression levels in fibroblasts 4 hours following IL-1*β* and TNF-*α* stimulation. Subsequent analysis has confirmed that PTX3 mRNA expression is elicited in a variety of cell and tissue sources following stimulation with IL-1*β*, TNF-*α*, and the proinflammatory stimulus lipopolysaccharide (LPS) through activation of the nuclear factor *κ*B (NF-*κ*B) and activator protein 1 (AP-1) transcription factors [32, 33], including the lungs, ovaries, thymus, brain, skeletal and cardiac muscle, visceral and subcutaneous adipocytes, hepatocytes, monocytes and macrophages, dendritic cells, and neutrophils [7, 8, 11, 34–38]. In addition, intravenous injection of LPS results in the elevation of systemic PTX3 concentrations in both rodents and humans [32, 35] due to activation of the PTX3 promotor genes within the cell. The presence of a leader peptide of 17 amino acids located in the N-terminus is essential for the secretion of protein [31]. Therefore, compared to CRP, the local expression and concomitant systemic increase of circulating PTX3 that occurs transiently in response to proinflammatory stimulation suggests that PTX3 may be a more sensitive and viable biomarker of tissue pathology and disease status.

## 3. PTX3 Dysregulation in Obesity

### 3.1. Obesity-Mediated Inflammation and Insulin Resistance

Obesity is defined as the excess accumulation of intra-abdominal body fat which can be subdivided into two compartments: subcutaneous adipose tissue (SAT) and visceral adipose tissue (VAT). While both tissue types are important, evidence suggests that VAT is a stronger predictor of obesity-related inflammation and metabolic disease [39, 40]. SAT and VAT accumulation is a result of the increased number and size of adipocytes [41, 42]. Analysis of adipocyte kinetics indicates that the number of adipocytes increases more dramatically in obese compared to that in normal-weight individuals throughout childhood and plateaus upon reaching adulthood, implying that further fluctuations of adipose cell mass are due to increased adipocyte hypertrophy resulting from excess lipid storage [41].

Adipose tissue is not just a passive storage site for fat accumulation but an endocrine organ contributing to the production of anti-inflammatory and proinflammatory proteins [43]. Adipocyte tissue consists of a mature adipocyte fraction and a stromal-vascular fraction (SVF) comprised primarily of preadipocytes, vascular cells, and leukocytes, including neutrophils and macrophages [44]. More specifically, neutrophils are rapidly recruited to the SVF in response to acute, high-fat challenge (i.e., diet), with peak concentrations occurring between 3 and 7 days [1]. This acute infiltration of neutrophils precedes the increased number of resident macrophages that accumulate within the SVF in response to as little as 8 weeks of high-fat diet [1]. Macrophages are a heterogeneous cell population which are roughly divided into two primary phenotypes, *classically activated* (M1) and *alternatively activated* (M2), based on their function and secretory characteristics and are identified by their unique array of cell surface markers [45, 46]. Macrophage phenotypes are largely determined by the microenvironment in which they reside [46, 47]. For example, local concentrations of the proinflammatory mediators LPS and interferon gamma stimulate the polarization of M1 macrophages that are responsible for the initiation of debris clearance and the production of the proinflammatory cytokines IL-1*β*, IL-6, and TNF-*α*, and the chemokine monocyte chemoattractant protein 1 (MCP-1) [48]. Conversely, M2 macrophage polarization is the result of IL-4 and IL-13 signaling [49]. In turn, M2 macrophages produce the anti-inflammatory cytokines IL-10 and TGF-*β*, which attenuate the proinflammatory response and initiate tissue repair [49, 50].

During obesity, excess VAT hypertrophy is associated with an increased concentration of macrophages [3]. In fact, some estimates suggest that macrophages account for up to 40% of the total nuclei content in adipose tissue of obese individuals, compared to only 10% in that of normal-weight individuals [3]. Furthermore, macrophages in obese individuals are polarized toward an M1 phenotype and express elevated levels of proinflammatory cytokine mRNA [3, 47]. To the contrary, resident adipose tissue macrophages in normal-weight individuals are primarily comprised of M2 macrophages which help the cellular response counterregulates a proinflammatory challenge [47]. Interestingly, the distribution of macrophages is also different in obese compared to that in normal-weight individuals. Weisberg et al. [3] demonstrate that macrophages in adipose tissue of lean mice are uniformly small and widely dispersed among the adipocytes, whereas macrophages from obese rodents are larger and gathered in aggregate. More recent studies have shown that over 90% of resident macrophages are localized around adipocytes that have died from lipid-induced cellular stress (up to 15 per dead adipocyte) [39, 51]. Although visceral adipocytes are about 20% smaller than subcutaneous adipocytes, their capacity to expand is less, contributing to an elevated rate (30-fold) of adipocyte cell death that results in the localization of M1 macrophages in obese individuals [39, 51].

The excess accumulation of M1 macrophages within VAT is now understood to be the primary source of circulating proinflammatory cytokines IL-1*β*, IL-6, and TNF-*α* that contribute to the state of chronic, low-grade inflammation observed during obesity [3, 4]. M1 macrophages also express and secrete the proinflammatory chemokine MCP-1 in proportion to the amount of VAT, resulting in the enhanced recruitment of circulating monocytes [52]. Bories et al. [46] further report that the systemic proinflammatory environment observed during obesity predisposes circulating monocytes toward a proinflammatory phenotype, and upon entry into the adipose tissue, monocytes migrate to the signaling site and preferentially differentiate into resident M1 macrophages. These findings suggest that the accumulation of M1 macrophages and increased concentrations of macrophage-derived proinflammatory proteins result in a positive feedback loop that further exacerbates the local and systemic proinflammatory milieu observed during obesity.

Studies also demonstrate that obesity-related proinflammatory as a result of M1 macrophage accumulation within VAT is associated with the number of metabolic syndrome parameters [3, 53] and is directly linked with the development and pathology of insulin resistance [4]. More specifically, macrophage-derived MCP-1, TNF-*α*, and to a lesser extent IL-6 directly impair the mechanisms involved with insulin-mediated glucose uptake in adipose tissue and skeletal muscle [4, 54–58]. However, neutralization of TNF-*α* and MCP-1 and the downregulation of proinflammatory cytokine production restore insulin-mediated glucose uptake in obese individuals [58–61] and suggest that targeting the production of macrophage-derived proinflammatory may positively regulate metabolic signaling.

### 3.2. PTX3 Expression in Obese Adipose Tissue

Abderrahim-Ferkoune et al. [7] document that TNF-*α* elevates PTX3 mRNA expression in the SVF but not the mature adipocyte fraction of adipose tissue in a dose-dependent manner. In addition, Osorio-Conles et al. [11] demonstrate that IL-1*β*, but not IL-6, contributes to PTX3 mRNA expression in adipose tissue. In parallel with these findings, adipose tissue expression of PTX3 mRNA is increased in obese compared to that in normal-weight mice and is 3-fold higher in diabetic-obese compared to that in nondiabetic obese mice [7]. These findings suggest that disease pathology may further enhance the expression of PTX3 mRNA in adipose tissue; however, whether or not this is due to metabolic dysregulation or the severity of proinflammatory status remains unknown.

In humans, VAT expression of PTX3 mRNA is positively associated with body mass index (BMI) [10] and is elevated in obese and overweight (BMI > 25 kg/m^2^) compared to that in normal-weight individuals (BMI 18.5–24.9 kg/m^2^) [11]. Furthermore, a positive association is observed between PTX3 and IL-1*β* mRNA expression in VAT, as well as TNF-*α* mRNA expression in SAT and VAT [10, 11]. However, greater PTX3 mRNA expression is reported in the mature adipocyte, compared to the SVF [11]. In support of these findings, Alberti et al. [10] report that PTX3 expression in adipose tissue does not appear to follow the pattern of macrophage distribution, as PTX3 mRNA expression is associated with the macrophage-specific surface marker CD68 in SAT, but not VAT, despite normal macrophage distribution (VAT > SAT; obese > normal weight). These findings suggest that adipose tissue components other than macrophages may be responsible for the elevated expression of PTX3 mRNA observed in obese humans.

In addition to inflammation, hyperinsulinemia, hypoxia, and reactive oxygen species are linked to the dysregulation of adipose tissue protein expression. However, human adipocyte PTX3 mRNA expression is only slightly responsive to hypoxic conditions and the mitochondrial complex II inhibitor antimycin [11], whereas H_2_O_2_ exposure results in a slight downregulation of PTX3 mRNA, and insulin stimulation has no effect [11]. In addition, circulating low-density lipoproteins (LDL) are a predictor of PTX3 mRNA expression in SAT of nondiabetic men [62], and high-density lipoproteins (HDL), which also regulate PTX3 mRNA expression in endothelial cells [63], are an independent predictor of PTX3 mRNA expression in VAT [10]. Therefore, additional research aimed at investigating the impact of circulating cholesterol may provide insight into the factors that contribute to elevated PTX3 mRNA expression in adipose tissue during obesity.

### 3.3. Circulating PTX3 Concentrations during Obesity

Circulating PTX3 concentrations are negatively associated with numerous indices of obesity, including BMI, waist and hip circumferences, and visceral fat mass (11, 67, 71, 86, 87, 99, 103), and positively associated with muscle mass (19). Analysis of daily fluctuations of plasma PTX3 demonstrates that systemic PTX3 concentrations are more stable compared to CRP [64]. These observations have increased interest in utilizing plasma PTX3 concentrations as a biomarker to assess the severity of obesity-related inflammation and metabolic disease. In contrast to the PTX3 mRNA expression patterns observed in adipose tissue of obese individuals, reports on plasma PTX3 concentrations in obese populations and in those with metabolic dysregulation have yielded inconsistent results. The majority of studies report that plasma PTX3 concentrations are lower in obese individuals and those with metabolic dysregulation compared to that in normal-weight and metabolically healthy controls [11–13, 62, 65–69]. To the contrary, other studies report the opposite [70–72]; however, elevated PTX3 concentrations observed in these studies may have been altered by the presence of atherosclerosis which may differentially impact plasma PTX3 concentrations in obese individuals. In fact, atherosclerosis induces a robust proinflammatory response observed in both the vascular endothelium and systemic circulation, and the parallel increases in PTX3 expression and secretion may potentially serve as protective mechanisms against the enhanced progression of atherosclerosis and associated proinflammatory milieu [73, 74].

Importantly, circulating PTX3 concentrations are impacted following weight loss intervention. Specifically, short-term bed rest with caloric restriction (14 days) increases plasma PTX3 concentrations in association with decreased fat mass [75], while regression analysis demonstrates that a 1-kilogram reduction in body weight is associated with a 74 pg/mL increase in plasma PTX3 concentrations following a 6-week dietary restriction intervention [69]. Witasp et al. [69] further demonstrate that increased plasma PTX3 concentrations are associated with reduced body weight, BMI, and central adiposity over a 5-year observational period. Although changes in metabolic parameters were not reported in these studies, numerous studies report that lower resting PTX3 concentrations are negatively associated with circulating concentrations of proinflammatory cytokines, triglycerides, insulin, glucose, and the HOMA-IR index of insulin resistance [11–13, 65–67, 75] and incrementally lower with increased parameters of metabolic syndrome [65, 67]. Plasma PTX3 concentrations are also negatively related with the insulin response following intravenous and oral glucose administration [11], and as Escobar-Morreale et al. [76] demonstrate, plasma PTX3 decreases in response to oral glucose intake. However, it remains unknown whether or not PTX3 decreases as a consequence of metabolic dysfunction or if PTX3 provides a mechanism to protect against the development of disease. A recent study utilizing a diabetic-obese rat model reports that in addition to lower circulating PTX3 concentrations, reduced PTX3 mRNA expression is associated with the reduced expression of the GLUT4 glucose transporter in skeletal muscle [77]. While these findings were observational in nature, this study suggests that PTX3 may aid in the facilitation of glucose homeostasis and highlights the need for additional research aimed at investigating the mechanisms associated with PTX3-mediated glucose uptake.

## 4. PTX3 as an Anti-Inflammatory Protein

### 4.1. Neutrophil Synthesis, Storage, and Release of PTX3

The reasons for the paradoxical findings between adipose tissue expression and systemic PTX3 concentrations are unclear, and the tissue source responsible for regulating systemic PTX3 concentrations is currently unknown. It is known that maturing neutrophils, but not other polymorphonuclear cells (basophils and eosinophils), synthesize and store ready-made PTX3 within lactoferrin granules [8]. Interestingly, while mature peripheral neutrophils do not express PTX3 mRNA or appear to be the primary source of systemic PTX3 concentrations, mature neutrophils do release stored PTX3 in response to LPS and TNF-*α* activation [8]. These findings suggest that mature neutrophils are a reservoir for PTX3 storage prior to their release in response to proinflammatory challenge.

Evidence suggests that neutrophil counts and rates of activation are increased in obese individuals and in those with metabolic dysregulation [1, 2, 78, 79]. Given this, the reduced concentrations of circulating PTX3 in these populations may potentially reflect an impaired ability of neutrophils to either synthesize PTX3 throughout their maturation process or release stored PTX3 upon neutrophil activation, posing deleterious risks during periods of inflammatory challenge. Conversely, it is also possible that reduced PTX3 concentrations may reflect the increased release of PTX3 by neutrophils within adipose tissue or increased tissue uptake of PTX3 from circulation by a mechanism currently unknown.

A novel study by Deban et al. [80] demonstrates that PTX3 selectively binds the adhesion molecule P-selectin. Following inflammatory stimulation, P-selectin relocates to the cell surface where it interacts with P-selectin glycoprotein on leukocytes [81]. As a result, leukocytes are arrested at the cell surface, infiltrate into the tissue, and stimulate a heightened immune response [81]. Thus, the binding of PTX3 to P-selectin aids in the regulation of the innate immune response by limiting the cellular infiltration of neutrophils and monocytes [73, 80]. In addition, these findings support the posit that PTX3 regulates the proinflammatory milieu observed during obesity, potentially by attenuating the infiltration of leukocytes into the adipose tissue.

### 4.2. Systemic Anti-Inflammatory Effects of PTX3

In humans, plasma PTX3 concentrations are inversely related to circulating proinflammatory cytokines (IL-6 and CRP) and positively related with the anti-inflammatory cytokine IL-10 [75]. The initial studies investigating the role of PTX3 as a mediator of inflammation utilized transgenic animal models which overexpress PTX3. Specifically, researchers injected the PTX3 transgenic mice with LPS and observed that PTX3 mRNA expression was elevated in a variety of tissues, including the heart and skeletal muscle, but not the liver [82]. Following LPS injection, survival curve analysis further revealed that transgenic expression of PTX3 rescued mice from endotoxic shock and reduces symptoms of sepsis [82]. Furthermore, PTX3 transgenic mice possess elevated concentrations of the anti-inflammatory cytokine IL-10 and exhibit a more robust anti-inflammatory response within the first few hours following LPS infusion [82].

Knockout animal models have also been utilized to demonstrate the adverse consequences of low-PTX3 concentrations. For example, Norata et al. [74] demonstrated that PTX3 knockouts experience increased atherosclerotic lesion size in the aorta, which was associated with the elevated expression of proinflammatory cytokine mRNA and the excess accumulation of macrophage within the atherosclerotic plaque. In addition, increased risk of myocardial infarction and elevated concentrations of circulating proinflammatory cytokines, as well as excess neutrophil and macrophage accumulation are observed in PTX3 knockout mice [73, 83]. However, exogenous PTX3 administration reversed this phenotype [73, 83], indicating that PTX3 is necessary to regulate the appropriate immune response.

### 4.3. Cellular Anti-Inflammatory Effects of PTX3

At the cellular level, proinflammatory stimulation by LPS is mediated by the pattern recognition receptor toll-like receptor 4 (TLR4) [84]. TLR4 is a transmembrane receptor which contains an intracellular cytoplasmic region homologous to the IL-1 receptor termed the toll/IL-1R (TIR) domain [85]. Activation of TLR4 signaling by LPS is reliant upon the action of two cell surface accessory molecules: myeloid differentiation protein 2 (MD-2) and CD14 [86]. Following ligand binding, TLRs undergo conformational changes resulting in the further recruitment of the myeloid differentiation factor 88 (MyD88) and TIR-containing adaptor molecule (TRIF) intracellular adaptor molecules that mediate the production of proinflammatory cytokines and type I interferons, respectively [87, 88]. Not surprisingly, TLR4 expression is elevated in adipocytes and circulating leukocytes in obese individuals and contributes to the increased risk of metabolic dysregulation [89–91].

Recent studies have also begun to elucidate the anti-inflammatory capacity of PTX3 at the cellular level. For example, the PTX3 N-terminus binds MD-2 and inhibits TLR4 activation in neutrophils, resulting in the reduced inflammatory burden following fungal infection [15]. In addition, activation of macrophages with high concentrations of PTX3 (10 ng/mL) attenuates LPS-induced production of IL-1*β*, TNF-*α*, and MCP-1 by downregulating NF-*κ*B protein expression [14]. Furthermore, PTX3 increases the production of the anti-inflammatory cytokines IL-10 and TGF-*β* through the Akt- and p38-mediated pathways, respectively [14–16, 92]. Although the mechanisms responsible for the PTX3-mediated anti-inflammatory signaling have not been fully elucidated, evidence suggests PTX3 signaling acts through TLR2, 3, and 4, but not TLR9 engagement, and is dependent upon TRIF-mediated activation of the transcription factor interferon-regulated factor 3, but not NF-*κ*B [15, 92].

These findings suggest that PTX3 is a counterregulatory protein which preferentially facilitates an anti-inflammatory response by downregulating the production of neutrophil and macrophage-derived proinflammatory proteins and increasing the production of anti-inflammatory cytokines. Thus, PTX3 may also facilitate the polarization of adipocyte macrophages toward an M2 phenotype, as recently suggested in different tissue sources [93, 94]. Therefore, it is reasonable to speculate that therapeutic approaches (i.e., weight loss, regular physical exercise, and the potential development of pharmacological interventions) which elevate circulating PTX3 concentrations in obese individuals will help restore obesity-related inflammatory imbalances and shift the systemic and local inflammatory microenvironments to an anti-inflammatory milieu.

## 5. PTX3 Response to Aerobic Exercise

Aerobic exercise training is an effective therapeutic approach against obesity-related proinflammatory and metabolic dysfunction. In fact, aerobic exercise training reduces adipocyte size, elicits the polarization of macrophages toward an M2 phenotype, and lowers proinflammatory cytokine expression in adipose tissue [95, 96]. As a result, the elevated local and systemic anti-inflammatory profiles following aerobic exercise training are associated with improved glucose metabolism and protect against high-fat diet-induced insulin resistance [96, 97].

Acute aerobic exercise also enhances systemic PTX3 concentrations. Our laboratory and others report that plasma PTX3 concentrations are increased for up to 1 hour in response to a single bout of aerobic exercise [12, 13, 17, 64]. Furthermore, Nakajima et al. [64] demonstrate that exercise-induced plasma PTX3 concentrations are positively associated with the neutrophil activation marker myeloperoxidase and that intracellular neutrophil concentrations of PTX3 are reduced compared to resting values in response to exercise in an intensity-dependent manner. These findings suggest that neutrophils are a significant source of elevated PTX3 concentrations following acute aerobic exercise, potentially serving as a mechanism to regulate the exercise-induced neutrophil infiltration into the tissue. Similarly, regular participation in aerobic exercise augments circulating concentrations of PTX3 at rest and in response to acute exercise. More specifically, resting PTX3 concentrations are increased in aerobically trained compared to that in sedentary males [98], and cardiorespirtatory fitness levels (VO_2max_) predict the acute exercise-induced PTX3 response following submaximal and maximal bouts of aerobic exercise [12, 13]. In addition, plasma PTX3 concentrations increase after 8 weeks of habitual moderate aerobic exercise training in elderly men and women, and these increases are associated with elevated cardiorespiratory fitness levels (VO_2max_) and improved indices of cardiovascular function [99, 100].

Data recently collected in our laboratory also demonstrates that the capacity of acute exercise to increase plasma PTX3 concentrations is similar in obese compared to that in normal-weight individuals [12, 13], suggesting that a single bout of aerobic exercise is equally beneficial for obese and normal-weight individuals. Furthermore, in response to 12 weeks of aerobic exercise training, elevated concentrations of resting PTX3 in obese and overweight females were associated with reductions in BMI [101]. From these results, one might be tempted to hypothesize that increased PTX3 concentrations may also mediate the anti-inflammatory effects of aerobic exercise in obese individuals. However, no associations have been observed between systemic PTX3 concentrations and indices of insulin resistance or proinflammatory following acute aerobic exercise in our laboratory [12, 13], suggesting that the cumulative effects of exercise training may be responsible for enhancing the anti-inflammatory effects of PTX3.

Unfortunately, only one study has investigated the relationship between PTX3 and indices of metabolic dysfunction in response to physical activity intervention. As a result of short-term physical activity intervention (7 days) in obese adolescents, increased plasma PTX3 concentrations were associated with decreased concentrations of circulating insulin as well as the HOMA-IR index [67]. Although limited by short duration, these findings provide evidence to warrant additional research aimed at investigating the effects of acute and chronic aerobic exercise intervention on PTX3 concentrations in obese individuals.

## 6. Conclusion

The findings presented in this review demonstrate that PTX3 may be a potential target for demonstrating and understanding the mechanistic impact of various therapies that address obesity-related chronic inflammation and metabolic dysfunction. With the growing obesity epidemic and the long-term health consequences of proinflammatory diseases, additional research focusing on the anti-inflammatory capacity of PTX3 against inflammatory-mediated metabolic dysfunction and the extent to which interventions such as weight loss programs aerobic exercise, and pharmacological agents may augment that these responses are necessary and warranted.

## Conflicts of Interest

The authors declare that there is no conflict of interest regarding the publication of this paper.
